# A public dataset of treadmill walking kinematics and kinetics at steady speeds in healthy individuals

**DOI:** 10.1016/j.dib.2026.112943

**Published:** 2026-06-06

**Authors:** L. Le Goff, F. Chorin, N. Reneaud, D. Fontaine, E. Piche

**Affiliations:** a Department of Physical Medicine and Rehabilitation, Princess Grace Hospital, Monaco; b UR2CA, Côte d’Azur University, Nice, France; c Clinique Gériatrique du Cerveau et du Mouvement, University Hospital of Nice, Nice, France; d LAMHESS, Côte d'Azur University, Nice, France; e Department of Physical Medicine and Rehabilitation, University Hospital of Nimes, Le Grau-du-Roi, France; f Department of Neurosurgery, Université Côte d'Azur, CHU de Nice, FHU INOVPAIN, Nice, France

**Keywords:** Gait analysis, Motion capture, Ground reaction forces, Locomotion biomechanics, Normative reference, Longitudinal assessment

## Abstract

Human motion analysis is widely used in clinical practice to characterise motor function, gait impairments, and fall risk. However, available gait datasets are predominantly acquired at self-selected walking speeds and rarely include standardised slower speeds, despite their relevance for clinical populations. This article describes a publicly available dataset of three-dimensional gait kinematics and kinetics collected in healthy adults under controlled treadmill conditions.

Forty-five participants, including young and older adults, performed walking trials at six predefined speeds ranging from 0.6 to 1.6 m·s⁻¹. Data acquisition was conducted using an optoelectronic motion capture system combined with an instrumented treadmill. The dataset includes three-dimensional marker trajectories, pelvis and lower limb joint kinematics in the sagittal, frontal, and transverse planes, as well as ground reaction forces, joint moments, and joint power. Raw data are provided in C3D format, and processed data are available as MATLAB-compatible files containing time-normalised gait cycles with associated mean values and variability metrics.

The dataset is structured to allow comparisons across walking speeds and age groups, with consistent acquisition protocols and standardised processing pipelines. Supplementary files include statistical descriptors and detailed biomechanical variables across the gait cycle. In addition, a composite metric derived from gait kinematic data across multiple speeds is provided to support integrative analyses of gait patterns.

This dataset can be reused for methodological development, validation of biomechanical models, and comparative analyses in both research and clinical contexts. The inclusion of standardised slow walking speeds and age-stratified data enables its use as a reference framework for evaluating gait patterns under conditions commonly encountered in clinical populations, including longitudinal assessments and comparisons with altered locomotor function.

Specifications Table

**Table:** 

Subject	Health Sciences, Medical Sciences & Pharmacology
Specific subject area	Three-dimensional gait biomechanics at six standardised walking speeds including slow locomotion
Type of data	Table; Figure; Graph; Raw; Processed; Filtered; C3D files; MAT files; XLS files
Data collection	Data were acquired using a 9-camera optoelectronic motion capture system (OptiTrack Motive 2.2.0, NaturalPoint, USA) at 100 Hz and an instrumented dual-belt treadmill (Bertec, USA) at 1000 Hz. Forty-five healthy adults walked barefoot at six predefined speeds (0.6–1.6 m·s⁻¹). Marker trajectories were filtered (6 Hz), ground reaction forces (20 Hz), and processed in MATLAB and OpenSim 4.1 using a scaled musculoskeletal model. Exclusion criteria included neurological or orthopedic disorders.
Data source location	University Côte d’Azur, Nice, France
Data accessibility	Repository name: Mendeley Data Data identification number: 10.17632/3dmymnb65h.1 Direct URL: https://data.mendeley.com/datasets/3dmymnb65h/1 Instructions for accessing these data: The dataset is available via the DOI link provided above. The code used for data processing and biomechanical analysis is publicly available on GitHub: https://github.com/LEGOFFL/Code-public-dataset-of-treadmill-walking-at-steady-speeds-in-healthy-individuals
Related research article	None

## 1 Value of the Data

• This dataset provides a comprehensive collection of three-dimensional gait kinematics and kinetics in healthy adults across six standardised treadmill walking speeds (0.6 to 1.6 m·s⁻¹), including slower speeds that are underrepresented in existing datasets. It includes both young and older individuals, along with raw (.c3d) and processed (.mat) data, enabling detailed biomechanical analyses across the full gait cycle.
• The dataset includes gait data collected at six predefined walking speeds, allowing comparisons under controlled and reproducible conditions. Given the strong influence of walking speed on gait biomechanics, the availability of multiple standardised speeds enables more appropriate matching between reference and individual data, particularly when self-selected walking speeds differ across subjects. The dataset can be reused for the development and validation of computational models, including musculoskeletal simulations, machine learning algorithms, and signal processing methods. The availability of synchronised kinematic and kinetic data supports advanced modelling approaches and methodological developments in biomechanics.
• The inclusion of both young and older adults allows the dataset to be used for age-stratified analyses. Researchers can explore differences in gait patterns across age groups and use these data as reference values for studies involving ageing populations or age-related functional changes.
• The dataset may be used as a reference framework for longitudinal and interventional analyses, including pre- and post-intervention comparisons. The availability of multiple walking speeds allows researchers and clinicians to select appropriate reference conditions when evaluating changes in gait performance over time.

## 2 Background

Quantitative gait analysis is increasingly used in clinical and research settings to characterise locomotor function using three-dimensional kinematic and kinetic measurements. These analyses rely on motion capture systems and force platforms to provide objective biomechanical data across the gait cycle and are widely used to evaluate gait impairments, monitor functional changes, and support clinical decision-making [[1], [2], [3]]. However, the interpretation of gait data relies on the availability of appropriate normative reference datasets. Existing gait datasets reported in the literature differ considerably according to the characteristics of the studied population (age, healthy individuals, or patients with specific conditions) and the methodological approaches used for gait assessment. These differences include walking conditions (overground or treadmill walking), walking speed (self-selected or imposed), distance covered, and the motion capture technologies or sensor systems used to record lower-limb movements. Consequently, the comparison and interpretation of gait data across studies remain challenging, underscoring the need for well-characterised normative reference datasets [4].

Walking speed is a major determinant of gait biomechanics, influencing lower-limb kinematics, joint moments, joint powers, and ground reaction forces. Consequently, meaningful comparisons between individuals or populations require consideration of walking speed. Nevertheless, many publicly available gait datasets rely on self-selected walking speeds, which vary across individuals and may reduce the reproducibility of biomechanical comparisons. Although several high-quality open-access gait databases have recently become available [[5], [6], [7], [8], [9], [10], [11]], few provide data acquired at identical predefined walking speeds for all participants. Furthermore, datasets including several standardised slow walking speeds remain scarce, despite the fact that reduced walking speed is commonly observed in older adults and in individuals with pain or locomotor disorders. Standardised speed conditions may therefore facilitate more meaningful comparisons between normative and clinical populations.

Age is another important factor influencing gait biomechanics. Older adults commonly exhibit alterations in joint mobility, propulsion, postural control, and medio-lateral stability compared with younger adults [12]. Consequently, reference datasets including both young and older adults are useful for distinguishing age-related adaptations from pathological gait deviations. While recent open-access datasets have substantially expanded the availability of normative gait data [[5], [6], [7], [8], [9], [10], [11]], datasets combining age-stratified populations with multiple predefined walking speeds, including clinically relevant slow speeds, remain limited.

This dataset provides raw and processed three-dimensional gait kinematics, kinetics, and ground reaction force data from healthy young and older adults walking barefoot on an instrumented treadmill at six predefined speeds ranging from 0.6 to 1.6 m·s⁻¹. By using identical speed conditions for all participants, including multiple slow walking speeds, the dataset provides a reproducible reference framework for speed-matched comparisons in both young and older healthy adults.

## 3 Data Description

The dataset is organised into two main folders corresponding to participant groups: “Young” and “Older”. Within each group folder, each participant is stored in an individual subfolder identified by a unique code (e.g., “01_LLG”, “02_NR”). Participant demographic and anthropometric information are provided in Table 1.

**Table 1:** Anthropometric information of included participants.

File name	Age (year)	Body mass (kg)	Height (cm)	Sex
01_LLG	33	72	178	Male
02_NR	28	70	173	Male
03_RC	25	79	184	Male
04_EP	26	60	169	Female
05_FC	37	99	189	Male
06_KS	24	76	176	Female
07_LD	26	70	179	Female
08_CF	46	55	159	Female
09_LV	29	60	160	Female
10_CF	28	72	167	Female
11_YD	23	65	182	Female
12_LF	23	50	165	Female
13_TP	30	87	198	Male
14_VP	36	65	176	Female
15_CB	28	60	172	Male
16_BC	63	58	160	Female
17_TC	60	82	182	Male
18_HJ	69	50	158	Female
19_AO	69	54	156	Female
20_OP	69	70	163	Female
21_JMM	64	63	172	Male
22_FM	66	65	162	Female
23_RLL	66	69	170	Female
24_RC	70	54	162	Female
25_MM	66	58	164	Female
26_EM	58	71	169	Female
27_VL	50	48	164	Female
28_AC	65	51	162	Female
29_MDV	65	45	160	Female
30_VD	64	77	165	Female
31_SZ	51	56	156	Female
32_AL	22	61	175	Male
33_MH	21	80	187	Male
34_AV	27	66	168	Female
36_AB	21	61	170	Female
35_DLG	68	75	180	Male
37_SD	21	66	173	Female
38_EPG	24	64	167	Female
39_XB	34	97	195	Male
40_VL	25	81	185	Male
41_MP	52	57	164	Female
42_CM	50	66	153	Female
43_FD	40	72	180	Male
44_PM	58	59	158	Male
45_MC	48	54	158	Female

Within each participant subfolder, seven raw data files in C3D format are provided. These include six walking trials corresponding to predefined treadmill speeds: “0_6.c3d”, “0_8.c3d”, “1_0.c3d”, “1_2.c3d”, “1_4.c3d”, and “1_6.c3d”, as well as one static calibration file named “static.c3d”. Each C3D file contains three-dimensional marker trajectories and synchronised ground reaction force signals. The structure of the force plate signals and analog channels is detailed in Supplementary Table 1.

Processed data are provided in MATLAB (.mat) format within the same participant subfolders. For each walking speed, one file is available (e.g., “0_60norm_data.mat”). Each file contains time-normalised data over 100 points of the gait cycle (0–100%) for both right and left sides. The variables include pelvis kinematics (“TILT”, “OBLIQUITY”, “ROTATION”), lower-limb joint kinematics (hip, knee, ankle), ground reaction forces (“Fx”, “Fy”, “Fz”), force plate moments (“Mx”, “My”, “Mz”), joint moments (“M_hip”, “M_knee”, “M_ankle”), and joint powers (“P_hip”, “P_knee”, “P_ankle”). Each variable is provided with mean values and variability using “mean”, “std_plus”, and “std_less”.

**Fig. 1:**
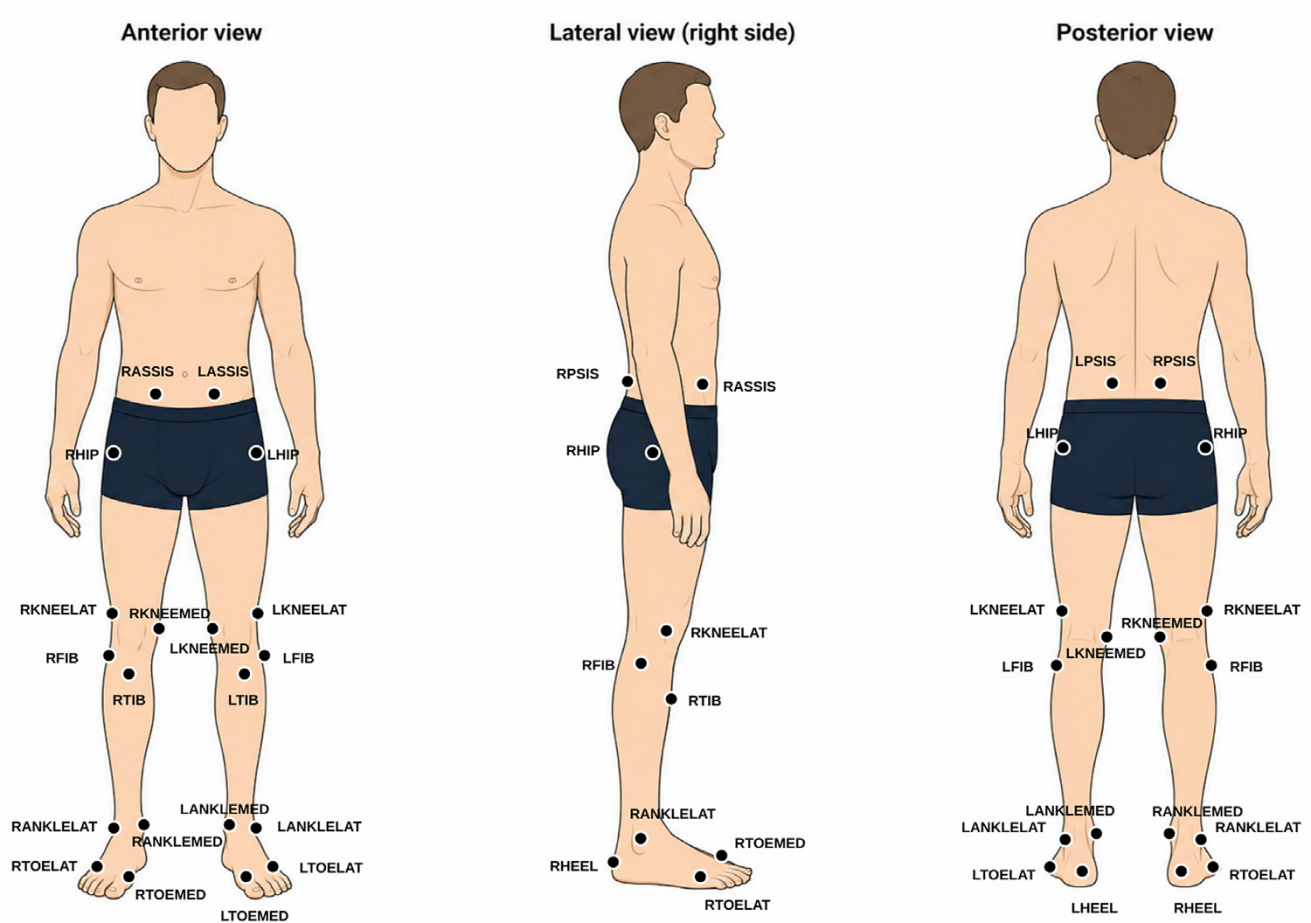
Marker-set protocol based on the modified IOR Leardini model.

Table 2 lists the anatomical location of the 24 reflective markers used for data acquisition and specifies the corresponding body segment or joint. Fig. 1 illustrates the marker-set protocol based on the modified IOR Leardini model [13] and provides a visual representation of the marker locations used to generate the kinematic variables contained in the dataset.

**Table 2:** Marker locations.

Marker name	Anatomical location	Segment or joint
LTOELAT	left head fifth metatarsal bone	left foot
RTOELAT	right head fifth metatarsal bone	right foot
LTOEMED	left head first metatarsal bone	left foot
RTOEMED	right head first metatarsal bone	right foot
LANKLELAT	left lateral malleolus	left foot
RANKLELAT	right lateral malleolus	right foot
LANKLEMED	left medial malleolus	left foot
RANKLEMED	right medial malleolus	right foot
LHEEL	left heel	left foot
RHEEL	right heel	right foot
LTIB	left tibial tuberosity	left knee
RTIB	right tibial tuberosity	right knee
LKNEELAT	left lateral condyle of femur	left knee
RKNEELAT	right lateral condyle of femur	right knee
LKNEEMED	left medial condyle of femur	left knee
RKNEEMED	right medial condyle of femur	right knee
LFIB	left head of fibula	left knee
RFIB	right head of fibula	right knee
LHIP	left great trochanter	left hip
RHIP	right great trochanter	right hip
LASIS	left anterior superior iliac spine	pelvis
RASIS	right anterior superior iliac spine	pelvis
LPSIS	left posterior superior iliac spine	pelvis
RPSIS	right posterior superior iliac spine	pelvis

Supplementary Table 2 contains statistical values (p-values and Cohen’s d) for 17 kinematic and kinetic variables across the gait cycle (1–100%) and for each walking speed.

## 4 Experimental Design, Materials and Methods

### 4.1 Participants

A total of 55 healthy adults were recruited. Due to a synchronisation module malfunction, complete datasets were available for 45 participants, which constitute the dataset provided in this repository. The final dataset includes 25 young adults (14 females, 11 males; mean age: 29 ± 7.5 years; mean height: 175 ± 11 cm; mean weight: 69.7 ± 12.4 kg) and 20 older adults (16 females, 4 males; mean age: 62 ± 6.8 years; mean height: 164 ± 7.5 cm; mean weight: 61.4 ± 10.2 kg) (Table 1).

All participants provided written informed consent prior to participation. The study protocol was approved by the local institutional review board (approval number: 2023-BS-546) and conducted in accordance with the Declaration of Helsinki.

### 4.2 Experimental setup

Data acquisition was performed using an optoelectronic motion capture system composed of nine infrared cameras (OptiTrack, NaturalPoint, Inc., USA) sampling at 100 Hz. Ground reaction forces were recorded simultaneously using a dual-belt instrumented treadmill (Bertec, Columbus, OH, USA) at a sampling frequency of 1000 Hz. The acquisition system was controlled using Motive software (version 2.2.0, NaturalPoint, Inc.).

A modified IOR Leardini marker set was used, consisting of 24 reflective markers placed on anatomical landmarks of the pelvis and lower limbs (Table 2) as illustrated in Fig. 1. Markers were positioned directly on the skin whenever possible or on tight-fitting clothing to minimise soft tissue artefacts.

### 4.3 Experimental procedure

Each participant completed a single acquisition session of approximately 30 min. Participants walked barefoot on the instrumented treadmill while wearing shorts and a fitted T-shirt. Prior to data collection, participants underwent a familiarisation period of at least 5 min to adapt to treadmill walking.

A static calibration trial was first recorded to define the biomechanical model. Participants then performed walking trials at six predefined speeds: 0.6, 0.8, 1.0, 1.2, 1.4, and 1.6 m·s⁻¹. For each speed, participants walked for 60 s, including 30 s of familiarisation followed by 30 s of data acquisition. Participants were instructed to walk naturally without holding the treadmill handrails.

### 4.4 System calibration and acquisition parameters

The motion capture system was calibrated prior to each session according to the manufacturer’s recommendations. This included definition of the global coordinate system, dynamic camera calibration, and zeroing of force plates. Marker trajectories were recorded in three dimensions and synchronised with ground reaction force data.

### 4.5 Data processing

Raw data were exported in C3D format and processed using MATLAB (MathWorks, USA). Marker trajectories and ground reaction forces were filtered using a fourth-order Butterworth filter with cut-off frequencies of 6 Hz and 20 Hz, respectively. A threshold of 20 N was applied to ground reaction forces to remove noise associated with treadmill artefacts.

Processed data were imported into OpenSim (version 4.1) for biomechanical modelling. The Rajagopal musculoskeletal model was used and scaled to each participant based on anthropometric measurements obtained during the static trial [14]. Inverse kinematics and inverse dynamics analyses were performed to compute joint kinematics, joint moments, and joint powers.

### 4.6 Time normalisation and signal processing

Gait cycles were identified based on ground reaction force thresholds. Data were segmented into individual gait cycles and time-normalised to 100 points (0–100% of the gait cycle). For each variable, mean values and variability were computed across cycles.

### 4.7 Software and code availability

Data processing and analysis were performed using MATLAB and OpenSim. Custom MATLAB scripts were used for signal processing, filtering, cycle detection, and data normalisation. These scripts are publicly available in the associated GitHub repository (https://github.com/LEGOFFL/Code-public-dataset-of-treadmill-walking-at-steady-speeds-in-healthy-individuals).

## Limitations

For one older participant, data are not available at the highest walking speeds (1.4 and 1.6 m·s⁻¹), as these conditions could not be completed during acquisition.

Data were collected during treadmill walking under controlled laboratory conditions, which differ from overground walking environments.

Although both young and older adults are represented, the sample size within each group remains limited.

Data acquisition and processing were performed using a specific motion capture system and musculoskeletal modelling framework, which may influence compatibility with datasets generated using different systems or modelling approaches.

## Ethics Statement

This study involved human participants. All participants provided written informed consent prior to data collection. The study protocol was approved by the local ethics committee (approval number: 2023-BS-546) and conducted in accordance with the principles of the Declaration of Helsinki.

## CRediT Author Statement

**Ludovic Le Goff:** Conceptualization, Methodology, Data curation, Formal analysis, Investigation, Writing - original draft preparation. **Frédéric Chorin:** Methodology, Supervision, Writing - review & editing. **Nicolas Reneaud:** Software, Data curation, Formal analysis. **Denys Fontaine:** Supervision, Writing - review & editing. **Elodie Piche:** Conceptualization, Methodology, Writing - original draft.

## Declaration of AI use in the Writing Process

During the preparation of this work, the authors used ChatGPT (OpenAI) to assist with language editing. After using this tool, the authors reviewed and edited the content as needed and take full responsibility for the content of the published article.

## Data Availability

Mendeley DataA public dataset of treadmill walking kinematics and kinetics at steady speeds in healthy individuals (Original data)

## Appendix Supplementary materials

Image, application 1

Image, application 2
