# Supplementary material for: A public dataset of treadmill walking kinematics and kinetics at steady speeds in healthy individuals
Source: Data Brief. 2026 Jun 6;67:112943. doi: 10.1016/j.dib.2026.112943 (PMC13273904; doi:10.1016/j.dib.2026.112943)
Supplement: Supplementary file 1 [file mmc1.docx]

| **Labels** | **Unit** | **Correspondant analog channel** | **Description** |
| --- | --- | --- | --- |
| Fx1 | N | Dev/ai161 | Force applied by the foot on plateform 1 component X |
| Fy1 | N | Dev/ai171 | Force applied by the foot on plateform 1 component Y |
| Fz1 | N | Dev/ai181 | Force applied by the foot on plateform 1 component Z |
| Mx1 | N.m | Dev/ai241 | Moment applied by the foot on plateform 1 component X |
| My1 | N.m | Dev/ai251 | Moment applied by the foot on plateform 1 component Y |
| Mz1 | N.m | Dev/ai261 | Moment applied by the foot on plateform 1 component Z |
| Fx2 | N | Dev/ai191 | Force applied by the foot on plateform 2 component X |
| Fy2 | N | Dev/ai201 | Force applied by the foot on plateform 2 component Y |
| Fz2 | N | Dev/ai210 | Force applied by the foot on plateform 2 component Z |
| Mx2 | N.m | Dev/ai271 | Moment applied by the foot on plateform 2 component X |
| My2 | N.m | Dev/ai281 | Moment applied by the foot on plateform 2 component Y |
| Mz2 | N.m | Dev/ai291 | Moment applied by the foot on plateform 2 component Z |
